# Reframing SpO_2_ tolerance as a physiological switch: implications for hypoxic adaptation and exercise regulation

**DOI:** 10.3389/fphys.2025.1667238

**Published:** 2025-09-03

**Authors:** Enomoto Yuri, Hui-Yu Chung, Fu-Shih Chen

**Affiliations:** ^1^ Graduate School of Pharmaceutical Sciences, Nihon Pharmaceutical University, Saitama, Japan; ^2^ Faculty of Pharmaceutical Sciences, Nihon Pharmaceutical University, Saitama, Japan

**Keywords:** SpO_2_ switch, physiological switch, hypoxia adaptation, autonomic nervous system regulation, threshold response, intermittent hypoxia training, SpO_2_ criticality

## Abstract

Blood oxygen saturation (SpO_2_) is a widely used oxygenation index in clinical and physiological settings. However, recent phenomena, such as asymptomatic hypoxia in COVID-19 and the superior performance of athletes in high-altitude conditions under hypoxia, have highlighted the significant variability in individual tolerance to blood oxygen saturation. Age, health status, disease, and hypoxic adaptation influence it. This brief review introduces the concept of the SpO_2_ switch as a dynamic. We also proposed a physiological compensatory response of SpO_2_ switch to SpO_2_ criticality that triggers compensatory responses, including ventilatory, autonomic, cardiovascular, and metabolic adaptations. Furthermore, individuals can exhibit markedly different responses to hypoxia at the same SpO_2_ value. It reflects a “threshold switch mechanism” driven by an individual’s internal physiological settings. This suggests that the SpO_2_ value demonstrates the onset of hypoxia symptoms and reacts to the body’s difference in compensatory capacity. This reconceptualisation shifts the focus from static thresholds to dynamic response analysis, offering new perspectives for precision health, mountain medicine, and personalised risk assessment of hypoxia.

## 1 Introduction

Oxygen saturation (SpO_2_) is a key indicator to assess respiratory and cardiovascular function (Swartz et al., 2020). Oxygen is essential for aerobic metabolism and maintaining cellular homeostasis (Trayhurn, 2019). The central respiratory control centers dynamically adjust breathing patterns and frequency in response to changes in arterial carbon dioxide (CO_2_) and oxygen concentrations (Urfy and Suarez, 2014). The nervous system is critical in voluntary and involuntary respiratory regulation (Cherniack, 1990; Health, 2022). Traditionally, SpO_2_ levels below 94% have been considered clinically alarming. However, during the COVID-19 pandemic, the phenomenon of “silent hypoxemia”—in which patients exhibit significant hypoxemia without overt symptoms—challenged traditional understandings of hypoxia and exposed limitations in current monitoring and critical care strategies (Dhont et al., 2020; Simonson et al., 2021; Bartlett et al., 2020; Yang et al., 2020).

Similarly, elite athletes and people living at high altitudes have excellent tolerance to low blood oxygen saturation (SpO_2_) levels. Systemic hypoxic stress increases as air pressure decreases with increasing altitude (Barnes and Kilding, 2015; Green, 2000). Hypoxic training has been used for a long time to enhance aerobic capacity by promoting adaptation to reduced oxygen availability (Sinex and Chapman, 2015). Since the outstanding performance of athletes from East African countries at the 1968 Mexico Olympics, altitude training has become a cornerstone of endurance training (Daniels, 1979; Jackson and Balke, 1971), Although hypoxic exposure can stimulate erythropoiesis, mitochondrial efficiency, and ventilatory responses, it can also impair performance in certain conditions (Sinex and Chapman, 2015).

There is growing interest in individual differences in hypoxic tolerance. Factors such as age, sex, genotype, history of altitude exposure, and ethnic background contribute to individual susceptibility to altitude-related illnesses, including acute mountain sickness (AMS), high altitude pulmonary edema (HAPE), and high-altitude cerebral edema (HACE) (Beall, 2014; Villafuerte and Corante, 2016). These differences are critical in designing altitude training programs and predicting adaptive responses (McLean et al., 2013).

Hypoxia is caused by a mismatch between oxygen supply and tissue metabolic demand (Maltepe and Saugstad, 2009). Of note, intense exercise under normoxic conditions also produces hypoxia-like responses due to the dramatic increase in oxygen demand (Radak et al., 2013). These responses span cognitive, visual, emotional, motor, and autonomic domains, and are influenced by physiological status, stress reactivity, exposure duration, and altitude, resulting in substantial interindividual variability (Asshauer, 2006). Although molecular biomarkers for predicting hypoxia tolerance have been explored, no reliable pre-exposure markers have been validated in humans or animal models (Dzhalilova and Makarova, 2020). Furthermore, ventilatory parameters such as tidal volume or respiratory rate may not fully capture the core drivers of respiration (Mortola, 2019).

These observations prompt reevaluating how SpO_2_ thresholds function and why individual tolerance varies. In this context, we introduced the concept of SpO_2_ dependence as a physiological switch that describes how changes in metabolic and ventilatory compensation shape individual hypoxic responses. This “switch” is a threshold-triggered response mechanism, indicating that SpO_2_ tolerance is not static, but can be dynamically adjusted and hierarchically trained.

Notably, even at similar or similar SpO_2_, individuals exhibit significant variability in their responses to hypoxia symptoms. Some people rapidly experience symptoms like dizziness and dyspnea, while others experience little to no symptoms. This phenomenon suggests that there may be an adjustable physiological threshold or “switch mechanism” that determines when to initiate the hypoxic compensatory response.

## 2 Individual differences in SpO_2_ tolerance

Individual tolerance to SpO_2_ varies significantly and is influenced by multiple factors, including age, physical condition, chronic diseases, genetics, and ethnic background.

• Age Factor

In healthy adults, resting SpO_2_ remains between 97% and 99%, with values below (Ceylan et al., 2016; Collins et al., 2015). SpO_2_ tends to decline with aging. Studies have shown that the mean arterial oxygen partial pressure (PaO_2_) in people over 80 years of age is approximately 66 mmHg, corresponding to an SpO_2_ of approximately 90%–92% (Malmberg et al., 1987; Sorbini et al., 1968; Cerveri et al., 1995; Madan, 2017).

• Chronic Disease Factors

Resting SpO_2_ values in patients with chronic diseases, including diabetes (Laursen et al., 2022), chronic cough (Sumanto and Ningtyas, 2022), chronic obstructive pulmonary disease (COPD) (Furian et al., 2018), and COVID-19 infection (Dhont et al., 2020; Simonson et al., 2021; Fuglebjerg et al., 2020), often range from 88% to 92%.

• Fitness and Training Status

Well-trained athletes typically have a delayed and smaller physiological response to decreased SpO_2_. During intense exercise, individuals often maintain elevated SpO_2_ levels (Rojas-Camayo et al., 2018; Eroglu et al., 2018; Martín-Escudero et al., 2021). Furthermore, individuals who engage in long-term high-altitude training, even with low resting SpO_2_, demonstrate high efficiency of their cardiopulmonary and oxygen transport systems (Rojas-Camayo et al., 2018).

• Ethnic and social factors

Ethnic differences may influence the clinical assessment and treatment strategies for hypoxemia. For example, oxygen therapy regimens in intensive care units vary across ethnic groups, and pulse oximetry may underestimate hypoxemia in patients with darker skin (Giovanelli et al., 2023; Sjoding et al., 2020; Fawzy et al., 2022). Furthermore, genetic background (such as high-altitude acclimatization; (Beall, 2007; Nishimura et al., 2022), access to healthcare, and socioeconomic status (Shi et al., 2022) also influence the diagnosis and prognosis of hypoxemia.

In summary, the triggering of hypoxic symptoms depends not only on the absolute SpO_2_ value but also on the individualised “SpO_2_ threshold switch.” In other words, even at the same blood oxygen concentration, different individuals may exhibit completely different symptomatic responses or no symptoms due to different threshold settings.

## 3 Physiological mechanisms of hypoxic compensation

When the body senses hypoxia, it initiates a series of compensatory mechanisms to maintain oxygen homeostasis, including increased respiratory rate, heart rate, sympathetic nerve activity, and redistribution of blood flow to vital organs (Grimminger et al., 2017). These responses are mainly mediated by chemoreceptors, especially those in the carotid arteries and aortic bodies, which can sense the decrease in arterial blood oxygen and trigger downstream physiological pathways (Prabhakar et al., 2015; Prabhakar and Semenza, 2015; Heymans and Heymans, 1927).

The autonomic nervous system (ANS) plays a central role in hypoxic adaptation. Increased sympathetic nervous system activity enhances cardiac output and pulmonary ventilation, while parasympathetic nervous system activity is typically suppressed to support the acute stress response (Hainsworth et al., 2007; Schagatay et al., 2000). Respiratory centres within the brainstem are highly sensitive to hypoxia and rapidly initiate a hypoxic ventilatory response (HVR) to increase ventilation and partially compensate for decreased blood oxygen levels (Pamenter and Powell, 2016). Prolonged hypoxia can cause a shift in baseline autonomic function, and individual differences in this response are closely related to genetic background, physical status, age, and sex (Puri et al., 2021). Previous studies have shown that exercise training can help improve autonomic stability, enhancing hypoxic tolerance (Calbet et al., 2003).

Acute hypoxia causes a decrease in arterial oxygen content, affecting multiple physiological functions. Under moderate hypoxic conditions, peripheral muscles are prone to fatigue and inhibit motor output through sensory afferent centres to reduce energy expenditure and maintain physiological stability. This is also one of the core assumptions of the “perception-limited fatigue theory” (Amann et al., 2006; Amann et al., 2007; Gandevia, 2001). Under more severe hypoxic conditions, even if muscles have not reached maximal fatigue, the body will actively reduce exercise output to avoid systemic instability (Fulco et al., 1994).

Under constant perceived exertion (RPE) conditions, exercise intensity and duration decrease significantly as ambient oxygen concentration decreases. This phenomenon is closely associated with a rapid decrease in SpO_2_ and a premature increase in respiratory rate, indicating that SpO_2_ levels and respiratory compensation are important physiological signals regulating perceived exertion (Jeffries et al., 2019). Exercise-induced hypoxemia still significantly limits aerobic capacity (Faoro et al., 2017). Low baseline SpO_2_ at rest is a significant risk factor for severe exercise-induced desaturation (EID) (Gao et al., 2025).

There is also significant inter-individual variability in ventilatory responses to intense exercise, which is difficult to predict using resting hypoxic or hypercapnic stimulation tests. Previous literature has generally suggested that trained endurance athletes exhibit blunted chemoreceptor responsiveness, but this phenomenon is highly heterogeneous and may be related to baseline SpO_2_ (Dempsey and Wagner, 1999).

At high altitude, the decrease in ambient oxygen partial pressure with increasing altitude naturally causes SpO_2_ to decrease. Despite this, most healthy adults can acclimate within hours to days, maintaining arterial oxygen saturation (SaO_2_) within the functional range of 80%–90% (Shaw et al., 2021). In contrast, elderly individuals exhibit blunted respiratory and cardiovascular responses to hypoxia and hypercapnia, suggesting that their oxygen dependence may increase (Kronenberg and Drage, 1973). Elderly individuals and those with chronic medical conditions are more affected by hypoxia-related symptoms and complications (Albert and Swenson, 2014; Chapman, 2013; Havalko et al., 2022).

Notably, an individual’s physiological response to hypoxia is highly related to their resting SpO_2_ level. Studies have shown that non-pharmacological interventions such as acupuncture may help improve hypoxemia-related symptoms by lowering SpO_2_ levels (Sumanto and Ningtyas, 2022). Intermittent hypoxia (IH) training is a non-pharmacological method for preventing and treating hypoxia in patients with various diseases and healthy adults (Serebrovskaya and Xi, 2016; Verges et al., 2015).

The extent and duration of the decrease in SpO_2_ at low oxygen doses (F(IO)_2_) can reflect an individual’s compensatory capacity. SpO_2_ levels remain stable in tolerant individuals, whereas SpO_2_ decreases rapidly and recovers slowly in dependent individuals, suggesting increased oxygen sensitivity (Peltonen et al., 1999). Furthermore, patients undergoing obesity surgery experienced elevated cardiopulmonary parameters and decreased SpO_2_ after a 6-min walk (Shrivastava, 2025). A study of sprinters undergoing high-intensity intermittent hypoxic training demonstrated that higher mean SpO_2_ levels were associated with improved performance, highlighting how changes in SpO_2_ influence training responses (Takei et al., 2025).

In summary, when the body senses hypoxia, it triggers a compensatory response through chemoreceptors, including increased respiratory and heart rates, sympathetic activity, and redistribution of blood flow to maintain oxygen homeostasis. The intensity of this response is influenced by genetics, age, physical fitness, and health status.

## 4 Regulation and adaptation of the SpO_2_ switch

Aerobic capacity—the ability to sustain prolonged exercise under normoxic conditions—is a key determinant of endurance performance (Feng et al., 2023; Girard et al., 2020). The brain and skeletal muscle have different oxygen requirements, and physiological or pathological states can alter tissue sensitivity to oxygen supply (Kulkarni et al., 2007). Although well-trained individuals typically have a low resting heart rate, they can still exhibit a pronounced heart rate response to hypoxic or high-intensity exercise (Goorakani et al., 2020; Patel and Zwibel, 2024).

Among various exercise training methods, interventions such as intermittent hypoxic training (IHT), breath-hold diving, and paced breathing exercises have significantly improved tolerance to low SpO_2_. These exercises can enhance autonomic balance (Rybnikova et al., 2022), ventilatory efficiency and metabolic regulation, oxygen transport and utilisation (Park et al., 2018; Park et al., 2018), and even exert neuroprotective effects (Rybnikova et al., 2022).

Intermittent hypoxia (IH) training, with the development and widespread use of equipment that induces systemic or localised hypoxia, has recently seen considerable research on related training methods. Methods such as “hypoxic living-hyperoxic training” have gained widespread popularity and become effective and efficient training methods for various professional athletes (Millet et al., 2010; Girard et al., 2017; Girard et al., 2020).

Well-trained freedivers can maintain a 1:1 apnea-to-repnea ratio while stationary without experiencing progressive hypoxia, and their physiological responses adapt with repeated pauses (Mulder et al., 2025). Furthermore, elite divers can tolerate prolonged apnea with minimal anaerobic metabolic burden (Drviš et al., 2025), suggesting that training strengthens the ability to regulate the SpO_2_ switch and prolongs tolerance. We believe this is due to the regulation of the SpO_2_ switch, resulting in adaptation after training.

In this study, arterial oxygen saturation was measured in healthy subjects and patients with chronic heart failure during spontaneous breathing, at 15, 6, and 3 breaths per minute, at rest, and during exercise (Bernardi et al., 1998). These exercises help maintain calmness and physiological stability under low oxygen pressure, supporting that spontaneous respiratory regulation can enhance autonomic function (Jerath, 2016). Even brief, conscious control of breathing rate and depth is considered a health-promoting strategy, similar to the mechanisms of altitude acclimatisation. In hypoxic emergencies, these techniques may help delay the onset of severe hypoxemia (Miles, 1964).

Acute hypoxia increases cardiac output and sympathetic drive to maintain oxygen delivery to vital organs (Heinonen et al., 2016; Fox et al., 2006). In severe COVID-19, the concurrent decrease in oxygen saturation and increased heart rate are associated with autonomic dysfunction or enhanced baroreflex sensitivity (Swenson and Hardin, 2023). Interestingly, despite metabolic changes under hypoxia, VO_2_ during fatigue was similar across normoxia, hypoxia, and hyperoxia, suggesting oxygen availability may not limit short-to moderate-duration exercise (Adams and Welch, 1980).

In summary, the best way to explain the varying manifestations of symptoms at the same SpO_2_ level is to view SpO_2_ as a dynamic physiological switch. Its individualised critical threshold (SpO_2_-CR) determines when compensatory responses are initiated.

## 5 Discussion

### 5.1 SpO_2_ switch: critical response range and regulation of hypoxia tolerance

SpO_2_ is commonly used to quantify oxygen transport status. However, recent studies suggest that a decrease in SpO_2_ can trigger a series of physiological compensatory responses, potentially acting as a “switch.” For example, high-altitude studies have shown that men with higher BMIs are more susceptible to hypoxemia during winter mountaineering (Vignati et al., 2021), and BMI is negatively correlated with SpO_2_ (Ceylan et al., 2016; Gupta et al., 2014). Obese subjects also have worse altitude sickness scores and nighttime SpO_2_ at a simulated altitude of 3,658 m (Ri-Li et al., 2003), reflecting limited respiratory acclimatisation and hypoxia tolerance (Caravedo et al., 2022). Furthermore, exercise testing has shown that a significant decrease in SpO_2_ shortens exercise time and reduces performance (Jeffries et al., 2019). Some non-pharmacological interventions, such as acupuncture, can also adjust SpO_2_ levels and alleviate hypoxia-related symptoms (Sumanto and Ningtyas, 2022).

In addition to high-altitude exposure, SpO_2_ during exercise also exhibits intensity-dependent characteristics. Cycling exercise studies showed that SpO_2_ after anaerobic exercise decreased significantly compared to before and after warm-up (Tahhan et al., 2018). Henslin Harris et al. (2013) and Campbell et al. (2009) noted that the decrease increased with increasing exercise intensity (Campbell et al., 2009; Henslin Harris et al., 2013); however, no significant changes were observed during warm-up or low-to-moderate-intensity aerobic exercise, SpO_2_ usually remains close to resting levels (Rowell et al., 1964). This may be because the respiratory and circulatory systems can maintain stability, keeping SpO_2_ close to resting levels (Tahhan et al., 2018).

Nikooie et al. (2009) used SpO_2_ to measure the anaerobic threshold (AT) noninvasively. They found that when exercise intensity reaches AT, SpO_2_ drops sharply and is highly correlated with the lactate threshold (LT), reaching its lowest point at maximal oxygen uptake (VO_2_max). Similar phenomena are observed in different types of exercise: for example, a rapid drop in SpO_2_ during the high-intensity phase can be observed in both short-distance, high-intensity anaerobic sprints (100 m) and medium- and long-distance aerobic events (400 m and 800 m).

**FIGURE 1:**
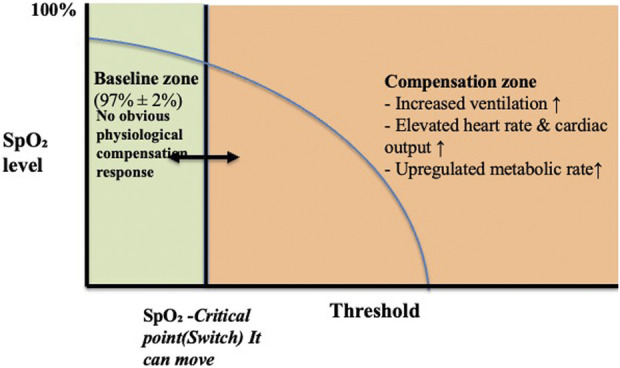
Physiological responses to declining SpO_2_: activation of the SpO_2_-CR switch. Note: When SpO_2_ remains within the normal range (approximately 97% ± 2%), the body is in a stable “baseline zone.” As SpO_2_ slowly decreases and approaches the individual’s critical point (But this varies from person to person), the SpO_2_-CR switch is triggered, entering the “compensatory zone.” Central and peripheral regulatory systems work together within this zone, including increased ventilation, heart rate, cardiac output, and metabolic rate.

These changes in SpO_2_ are not simply due to insufficient oxygen supply but result from coordinated regulation between the central and peripheral systems. This leads us to propose the “SpO_2_-CR switch” hypothesis: baseline SpO_2_ remains stable. When exercise intensity approaches VO_2_max, SpO_2_ drops to an individualised nadir, but does not deviate significantly from baseline. This “switch” may trigger the hypoxic response, determining the body’s compensation pattern under high load (see Figure 1).

Furthermore, the hypoxic threshold may vary among individuals. Modulating this threshold “switch” through medication, acupuncture, or other non-pharmacological approaches may further optimise hypoxia-related physiological responses and athletic performance.

These responses aim to maintain tissue oxygen delivery and exercise performance in hypoxic environments. When SpO_2_ rises and exceeds the critical point, the switch “resets,” and physiological functions gradually return to baseline levels.

It is important to note that the “baseline zone” and “critical point” are not fixed values but can be adjusted through training, environmental adaptation, and even pharmacological or non-pharmacological interventions. Training adaptation can lower baseline SpO_2_ levels or delay the triggering of the critical point, thereby improving hypoxic tolerance and exercise performance.

Based on this, we propose the concept of the SpO_2_ switch and critical range as individualised indicators for inducing compensatory responses. Its core components include:

1. Baseline SpO_2_: The average SpO_2_ range of an individual’s stable SpO_2_ at rest and normal pressure.
2. Critical Range (SpO_2_-CR): A certain drop below the baseline value is considered a threshold that may trigger a response.
3. Switch Activation: When SpO_2_ enters the critical range, compensatory mechanisms such as increased respiratory and heart rates, sympathetic nerve activation, and blood flow redistribution are triggered.
4. Trainability: Interventions such as breathing training, endurance exercise, high-altitude exposure, or acupuncture can adjust baseline and critical ranges to improve hypoxia tolerance.

This concept can be applied to athletic performance monitoring, chronic disease management, and altitude acclimatisation assessment. Future research could explore its feasibility as a clinical predictive and training indicator.

## 6 Future research directions and clinical applications

SpO_2_ should not be understood simply as a passive reflection of oxygen delivery but as a dynamic physiological switch that controls the body’s compensatory response to hypoxic stress. This switch influences the individualised SpO_2_ critical threshold (SpO_2_-CR). Below this threshold, the body initiates a series of adaptive mechanisms, including increased ventilation, increased heart rate, sympathetic nervous system activation, and redistribution of blood to vital organs. This switch-like behaviour of SpO_2_ has important implications for understanding exercise tolerance, fatigue, and resilience under both hypoxic and non-hypoxic conditions. It is expected to be a comprehensive physiological indicator encompassing multiple fields, including altitude acclimatisation, physiological monitoring, exercise training, and critical care.

Although previous research has explored the significance of SpO_2_ in clinical and environmental physiology, its regulation, modelling, and systematic validation remain limited.

Future research should explore various interventions to modulate the SpO_2_ switch. Breathing training, structured exercise in hypoxic conditions, and high-altitude exposure may help lower the critical threshold and enhance hypoxic tolerance. Furthermore, previous studies have shown preliminary efficacy in modulating SpO_2_ responses, particularly in individuals with irregular blood pressure or chronic respiratory symptoms, warranting further investigation as a non-pharmacological intervention. Pharmacological modulation of the SpO_2_ switch response also represents an emerging area, promising therapies to enhance oxygen utilisation or prevent hypoxic injury.

This approach could be applied to high-altitude travel, aviation medicine, geriatric care, sports training, and rehabilitation medicine to develop personalised health management and risk prevention strategies. Integrating genetic, epigenetic, and environmental exposure profiles can help better understand the cross-scale mechanistic integration of individual differences in hypoxic adaptation.

## 7 Conclusion

Blood oxygen saturation (SpO_2_) should not be viewed solely as a passive indicator of oxygen delivery. Instead, it acts as an active physiological switch, regulating the body’s compensatory response to hypoxic stress. This conceptual model redefines the SpO_2_ switch as a dynamic and trainable trait, determined by an individual’s baseline level and a critical threshold (SpO_2_-CR). When SpO_2_ levels fall below this personalized threshold, a series of compensatory mechanisms are activated to maintain physiological and functional stability.

This conceptual model redefines SpO_2_ tolerance as a dynamic and adjustable trait, offering new perspectives for preventive medicine and precision health. Moving beyond a static threshold model and toward a personalized SpO_2_ response model can enhance early intervention, optimize training outcomes, and improve human adaptability and resilience to various physiological and environmental challenges.

In summary, even at the same or similar SpO_2_ percentages, significant differences exist between individuals in their physiological and symptomatic responses to hypoxia. This variability reflects the individualized SpO_2_ switching mechanism, whose critical threshold (SpO_2_-CR) determines when to initiate respiratory and circulatory compensatory responses.
